# Adolescents Suffering from Long-term Cyberbullying Victimisation: Peer Pressure and Anger Dysregulation as Risk Factors

**DOI:** 10.1007/s10802-025-01339-5

**Published:** 2025-06-11

**Authors:** Esperanza Espino, Ana Margarida Veiga-Simão, Paula Costa Ferreira, Virginia Sánchez-Jiménez, Rosario Del Rey

**Affiliations:** 1 Department of Psychology, Universidad Loyola Andalucía, Dos Hermanas (Seville, Andalusia), Spain; 2 Faculty of Psychology, Universidade de Lisboa, Lisboa, Portugal; 3 Department of Developmental and Educational Psychology, Universidad de Sevilla, Seville (Andalusia), Spain; 4 Faculty of Educational Sciences, University of Seville [Universidad de Sevilla], Pirotecnia Street, Seville, 41013 Spain

**Keywords:** Cyberbullying, Victimisation, Persistence, Peers, Anger

## Abstract

Episodes of cyberbullying victimisation have serious consequences among adolescents, which worsen when their involvement is perpetuated over time. It is therefore important to understand what factors lead to long-term cybervictimisation to prevent it. This one-year longitudinal study examines significant socioemotional factors in the origin and dynamics of cybervictimisation, not yet jointly explored in its perpetuation. Participants were 427 Spanish 7th -, 8th -, 9th - and 10th -grade students (52.9% boys, 46.8% girls, 0.2% other), aged 12–17 (*M*_*age*_ = 13.08, *SD* = 1.01). Adolescents completed a series of self-reported questionnaires assessing peer pressure, anger dysregulation, and cybervictimisation. The results revealed that: (a) of the total sample, 5.6% were cybervictims only at T1 and 8.0% only at T2, and 3.6% were long-term cybervictims; (b) all variables were significantly and positively correlated at T1 and T2, except cybervictimisation at T1 and anger dysregulation at T2; (c) scores varied according to gender and age; and (d) peer pressure can increase the risk of long-term cybervictimisation, specifically in cases where anger dysregulation levels are moderate or low. The results highlight the importance of considering moderating mechanisms involved in increasing the risk of long-term cybervictimisation as well as the need to promote positive peer group dynamics and emotion management to avoid perpetuating the problem.

## Introduction

The international revision of the term ‘cyberbullying’, published by UNESCO Chair in 2024, highlighted that this type of relational violence through the electronic media is characterised by: (i) being a *damaging social process*, i.e. it occurs in a network of interpersonal relationships; (ii) under an *imbalance of power* driven by collective norms; (iii) which can be perceived *repeatedly*, either by the frequency of cyber-aggressions over time or by the severity of their negative effects; (iv) and which manifests itself as *unwanted behaviour* that often causes emotional and social harm, including in the long-term (UNESCO Chair, 2024). Among adolescents and young people, posting hurtful comments or rumours, whether once or several times, are the most common types of cyber-aggressions (Andrade et al., 2021), for example to make fun of the victim’s appearance. These cyber-aggressions can be perpetrated by one or more individuals from anonymous profiles and can be witnessed by a wide audience by being reposted or shared with others (Menin et al., 2021).

The extent of peer-to-peer cyberbullying is a public health concern with serious implications for a considerable proportion of adolescents and young people around the globe. A recent meta-analysis by Li and colleagues (2024) that included 42 studies from Europe, Australia, North America, Asia, and the Middle East, with 266,888 participants, found that the mean prevalence of cybervictims was 11.1%. Similarly, the results of a study with 12,285 Spanish adolescents aged 11–18 years showed that the prevalence of cybervictims over the past two months was 8.1% (González-Cabrera et al., 2020). Findings regarding gender and age differences in cybervictimisation are mixed. While some studies report higher rates among girls and from middle adolescence, e.g. ages 13–15 (Tsitsika et al., 2015; World Health Organisation [WHO], 2020), others have found no significant differences (Smith et al., 2019).

### A Longitudinal Perspective on Involvement in Cyberbullying Victimisation

Given the significant prevalence rates of cyberbullying, considerable research has focused on understanding its evolution and impact, particularly among cybervictims (Chu et al., 2018; Gámez-Guadix et al., 2015).

In the study of cybervictimisation trajectories, greater attention has been given to role changes – such as a cybervictim becoming a cyberbully – than to the persistence or chronification of cybervictimisation over time (Ak et al., 2015; Camacho et al., 2021). Moreover, when persistence has been examined, it has more often been in the context of traditional school bullying rather than cyberbullying (Calvete et al., 2018; Romera et al., 2020). The limited existing research on persistent cybervictimisation suggests that most cybervictims do not remain in this role consistently but instead tend to disengage (i.e. sporadic cybervictims) or shift roles over time (Festl et al., 2017). However, there is a small proportion of them who experience cyber-aggressions over prolonged periods of time, leading to more severe consequences and making intervention more challenging (Johander et al., 2023). For instance, González-Cabrera and colleagues (2023) found in their prospective study of 3 waves over 13 months that among 1,142 students aged 11–18, 6% were stable cybervictims throughout the entire period analysed (mostly girls, with 4.3%).

Despite growing interest in cyberbullying, the risk factors underlying long-term cybervictimisation remain largely underexplored. Few studies have examined this issue; some have identified depressive symptoms (Huang et al., 2022) and poor-quality friendships (Tian et al., 2023) as potential risk factors, while others point to traits like mindfulness as protective (Royuela-Colomer et al., 2018). Advancing research in this area is critical, as cybervictimisation is known to be associated with serious negative outcomes – including emotional distress, depression, impaired social functioning and suicidal ideation (Coelho & Marchante, 2018; van den Eijnden et al., 2014). It stands to reason that the longer the cybervictimisation persists, the more severe and entrenched these consequences are likely to become (González-Cabrera et al., 2020, 2023). Accordingly, this study examines peer pressure and anger dysregulation – two factors typically studied in isolation in relation to sporadic cyberbullying involvement – but not jointly in the context of long-term victimisation.

### The Effect of Peer Pressure in Cyberbullying Victimisation

‘Peer pressure’ is understood as the influence that others exert over one’s own thoughts or behaviour to prioritise group norms as a sign of social identity (Santor et al., 2000). Adolescents and young people today may be increasingly susceptible to such influence in both face-to-face and online interactions, where social identity is often constructed and validated by peers (Gámez-Guadix et al., 2013). While peer pressure has been linked to the perpetration of cyberbullying – particularly when aggressive behaviour is reinforced or encouraged by the peer group (Piccoli et al., 2020) – its role in cybervictimisation remains less explored. The few existing studies on peer pressure and cybervictimisation pointed out that this relationship is far from trivial (Livazovi’c & Ham, 2019). Longitudinal research has shown, for instance, that peer pressure can mediate the relationship between cybervictimisation and its negative psychological consequences (Gao et al., 2021). However, further theoretical and empirical work is needed to understand whether and how peer pressure may serve as a risk factor for the persistence of cybervictimisation over time.

From a theoretical perspective, perceiving high peer pressure may contribute to long-term cybervictimisation by reinforcing maladaptive peer norms that normalise or tolerate aggression, while simultaneously reducing the cybervictim’s ability or willingness to resist or disengage from harmful peer dynamics. In this sense, peer pressure can indirectly sustain continued cybervictimisation by compromising assertiveness and heightening sensitivity to social cues and approval. Supporting this, recent evidence by Onditi and colleagues (2024) in a cross-lagged longitudinal design found that lower resistance to peer pressure was associated with increased future cybervictimisation. This suggests that adolescents who feel unable to resist group expectations may be less likely to defend themselves, report abuse, or disengage from online environments that expose them to harm – thereby increasing their vulnerability over time.

In this vein, there is ample evidence that the desire for social connection and peer acceptance in digital contexts may increase exposure to hurtful judgments online (Andrade et al., 2021; Gámez-Guadix et al., 2013). Adolescents who have experienced cybervictimisation often express a heightened need for belonging, companionship, and restoration of damaged social status (Fang et al., 2020; Navarro et al., 2015; Romera et al., 2020). This need may drive them to stay connected in peer spaces where they continue to be targeted, especially if they fear exclusion or social isolation (Gao et al., 2021).

### The Effect of Anger Dysregulation in Cyberbullying Victimisation

Research on cyberbullying and anger is extensive. In relation to cybervictimisation, two complementary perspectives have emerged. Some studies suggested that anger is a defining trait among cybervictimised adolescents and young people who struggle to express this emotion in adaptive ways (Lonigro et al., 2014; Pratt et al., 2014). Others argued that anger often arises as a reaction to cyberbullying (den Hamer & Konijn, 2016), with cybervictims displaying poor impulse control and reactive behaviors (Camacho et al., 2021; Ortega et al., 2012;). Specifically, those who are cybervictimised may react with anger-out, i.e. aggressively, or with anger-in, i.e. by suppressing themselves (Ak et al., 2015). For instance, cybervictims are more likely to ruminate, which is itself an expression of anger (Liu et al., 2020; Navarro et al., 2015). Given that emotional dysregulation – particularly anger dysregulation – has been linked to both cyberbullying involvement and the development of psychiatric symptoms (den Hamer & Konijn, 2016; Zsila et al., 2019), it is important to explore whether it also contributes to the persistence of cybervictimisation over time. It is plausible that cyberbullies are reinforced by the dysregulated emotional reactions of their cybervictims, which could increase the likelihood of continued perpetration.

### The Present Study

While the combined role of peer pressure and anger has been previously examined in relation to cyberperpetration (e.g. Yang et al., 2023), their influence on cybervictimisation remains underexplored – particularly in long-term cases. Yang and colleagues (2023) found that trait anger predicted perpetration, especially among adolescents experiencing high peer pressure. This raises the question of whether similar mechanisms may also contribute to continued cybervictimisation. Existing evidence suggests that, independently, both conformity to peer pressure and poor anger management may act as maladaptive coping strategies, increasing vulnerability to cybervictimisation and exacerbating its consequences (Camacho et al., 2021; Gao et al., 2021). In this regard, we propose that cybervictims exposed to both high peer pressure and poor anger regulation are especially vulnerable to ongoing cybervictimisation over time (T1–T2). Peer pressure may undermine their assertiveness, while anger dysregulation can lead to reactive behaviours that provoke further cyber-attacks. When these factors co-occur, they may significantly impair cybervictims’ ability to cope, resist coercive dynamics, or seek support – ultimately reinforcing or worsening their cybervictim status. Moreover, the role of peer pressure on long-term cybervictimisation may depend on cybervictims’ levels of anger regulation, which could either amplify or buffer its influence (see Direct and Moderating Effects below).

**Fig. 1:**
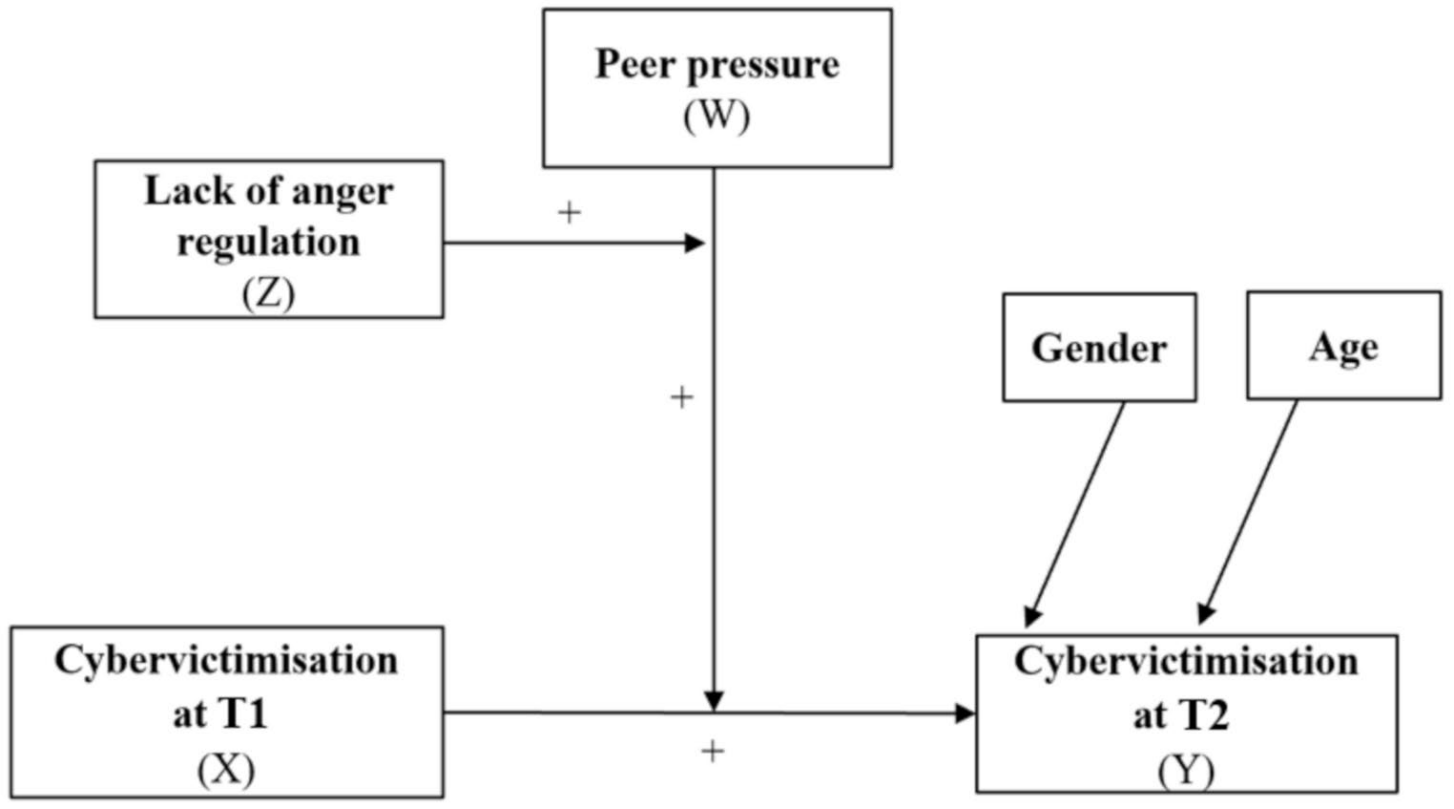
Theoretical model with two moderators *Note*. X = independent variable; Y = dependent variable; W = first moderator; Z = second moderator

Therefore, the present study explored whether peer pressure and anger dysregulation exert direct and moderating effects on cybervictimisation at Time 1 (T1) and one year later at Time 2 (T2), with the aim of clarifying their role in the persistence of cybervictimisation over time, while controlling for gender and age (see Fig. 1).

#### Direct Effects

**Fig. 2:**
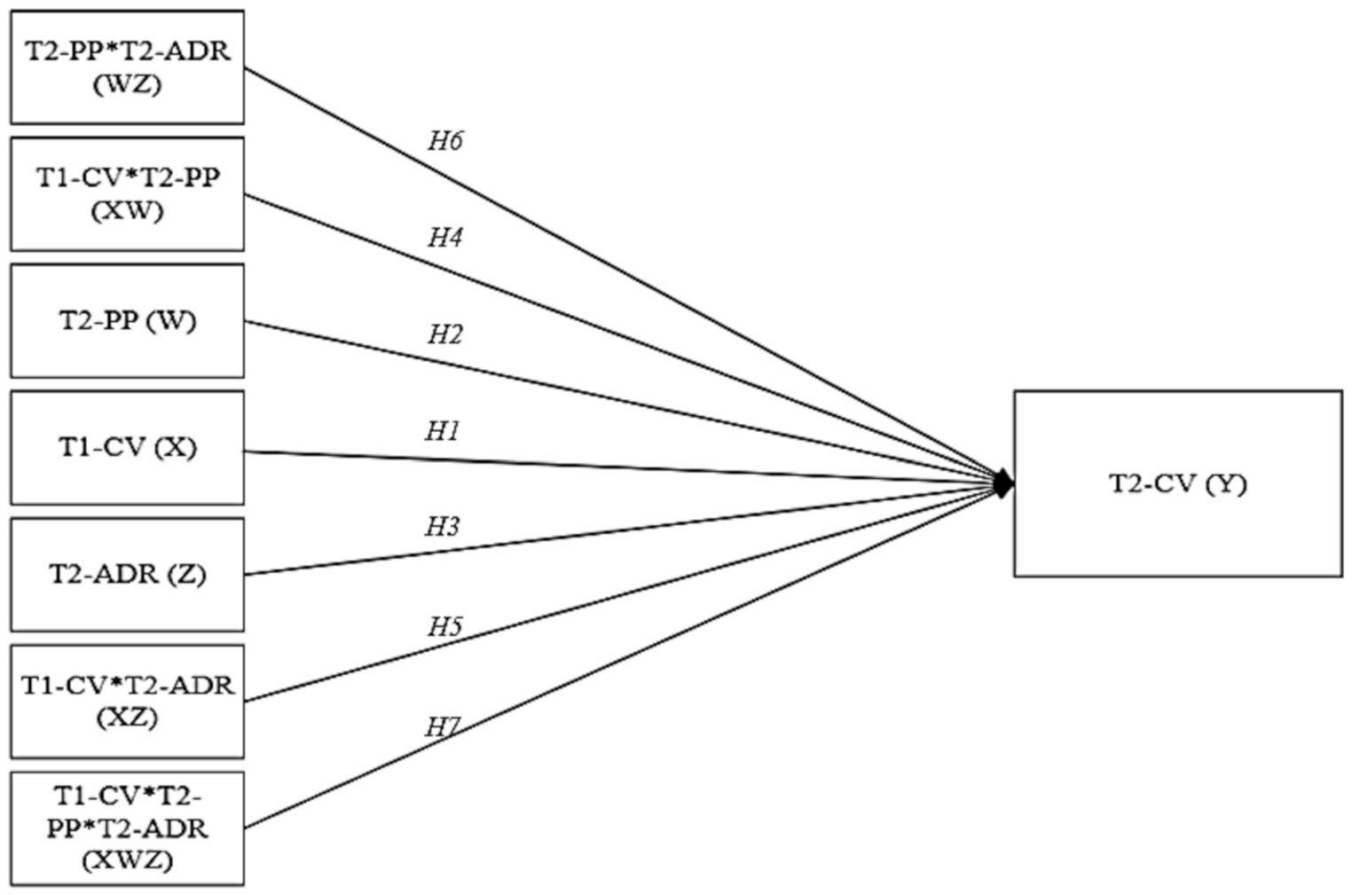
Statistical model with two moderators *Note*. X = independent variable (T1-CV = cybervictimisation at T1); Y = dependent variable (T2-CV = cybervictimisation at T2); W = first moderator (PP = peer pressure); Z = second moderator (T2-ADR = anger dysregulation); H = hypothesis

Regarding direct effects, we hypothesised: (H1) cybervictimisation at T1, (H2) peer pressure and (H3) anger dysregulation are positively associated with cybervictimisation at T2 (see Fig. 2).

#### Moderating Effects

Regarding moderating effects, we hypothesised: (H4) peer pressure and (H5) anger dysregulation can moderate the association between cybervictimisation at T1 and T2; (H6) anger dysregulation can moderate the association between peer pressure and cybervictimisation at T2; and (H7) anger dysregulation can moderate the conditional influence of peer pressure on the perpetuation of cybervictimisation (see Fig. 2).

## Method

### Participants

Participants were 427 Spanish secondary school students (52.9% boys, 46.8% girls, 0.2% other). Ages ranged from 12 to 17 years (*M* = 13.08, *SD* = 1.01 at T1, and *M* = 14.00, *SD* = 0.97 at T2). Regarding their school year at T1, 47.4% were in 7th grade, 42.3% in 8th grade, 9.9% in 9th grade and 0.5% in 10th grade. At T2, 3.3% were in 7th grade, 48.5% in 8th grade, 40.3% in 9th grade and 8.0% in 10th grade. To address the aim of this longitudinal study, only students who participated at both time points, T1 and T2, were selected in the main analysis. Hence, the percentage of eligible students that actually participated was 91% (for more details on the missing data analysis, see below).

### Procedure

The present study was approved by the Andalusian Ethical Coordination Committee for Biomedical Research, which is associated with the Virgen Macarena and Virgen del Rocío hospitals (code: 1223-N-18). To conduct a longitudinal study in which 12 months elapsed between two time points, a convenience sample was recruited from four secondary schools in southern Spain. As step 1, we explained and asked for the collaboration of the management teams by email and telephone. All secondary schools that confirmed their interest and willingness to participate in the longitudinal process were included. At step 2, the signed written consent of the families or legal guardians was requested, as well as the assent of the authorised students. They participated voluntarily and anonymously in a paper-and-pencil self-report survey during 15–20 min of class time and were supervised by teachers and our research team members at T1 and T2. Data were collected over the course of 2021.

### Measures

First, participants were asked about their gender, age and grade and, additionally, three validated scales were administered:

***Cyberbullying victimisation***. Involvement in cybervictimisation at T1 and T2 was measured using the victimisation subscale of the *European Cyberbullying Intervention Project Questionnaire*, *ECIP-Q*, validated for the Spanish population with optimal values (Ortega-Ruiz et al., 2016). This subscale includes 11 items referring to aggressions experienced over the internet and on social media sites in the last two months (e.g. ‘someone has insulted me’, ‘threatened me’, or ‘ignored me’). Responses are given on a 5-point Likert-type scale, from 0 ‘Never’ to 4 ‘Yes, more than once a week’. Good reliability values were obtained (Cronbach’s α_T1_ = 0.95; Cronbach’s α_T2_ = 0.94), along with optimal results in the confirmatory factor analysis (CFA) at T1 (χ² S − B = 113.93; DF = 44; *p* <.001; NNFI = 0.98, CFI = 0.99; RMSEA = 0.06, SRMR = 0.07) and T2 (χ² S − B = 157.04; DF = 44; *p* <.001; NNFI = 0.96, CFI = 0.97; RMSEA = 0.08, SRMR = 0.09).

***Peer pressure***. The influence of the group norms on participants’ behaviour was measured using the validated Spanish version of the *Extreme Peer Orientation* subscale of the *Peer Orientation Scale* by Fuligni and Eccles (1993) (see Sánchez-Jiménez et al., 2023). This one-dimensional scale includes 4 items referring to the perception of typical transgressive behaviour within peer group dynamics (e.g. ‘How many times have you done things which are outside your own capabilities in order to please your friends?’ or ‘How often do you disobey your family so that your friends won’t reject you?’), with responses being given on a 5-point Likert-type scale, from 0 ‘Never’ to 4 ‘Always’. An acceptable reliability value was obtained (Cronbach’s α_T2_ = 0.79), along with good results in the CFA at T2 (χ² S − B = 12.19; DF = 2; *p* <.001; NNFI = 0.94, CFI = 0.98; RMSEA = 0.11, SRMR = 0.04).

***Anger dysregulation***. Difficulties in controlling and expressing anger in stressful situations were measured using a subscale of the *Emotional Quotient Inventory: Youth version of* Bar-On and Parker (2000), already validated in Spanish population (see Méndez et al., 2019). This subscale includes 8 items referring to typical expressions of anger (e.g. ‘I fight with people’ or ‘When I get angry, I act without thinking’). Responses are given on a 5-point Likert-type scale, from 0 ‘Never’ to 4 ‘Always’, so high scores equate to poorer anger management. Good reliability was obtained (Cronbach’s α_T2_ = 0.87), along with acceptable results in the CFA at T2 (χ² S − B = 79.79; DF = 20; *p* <.001; NNFI = 0.96, CFI = 0.97; RMSEA = 0.09, SRMR = 0.06).

### Data Analysis

Data analysis was conducted using SPSS v.29 and EQS v.6.4. Prior to the analyses, data were coded and cleaned in SPSS v.29. Only participants who completed both T1 and T2 were included in the analyses. To assess the nature of the missing data, Little’s MCAR (Missing Completely at Random) test (Little, 1988) was performed, yielding a significant result (χ² = 2896.928; df = 2539; *p* <.001). However, the normed chi-square was low (χ²/df = 1.14) and adjusted for sample size sensitivity, which, according to Bollen’s (1989) criteria, supports the assumption that the data were missing at random (MAR). The overall response rate was 91% (*N* = 389).

Subsequently, reliability and validity analyses were carried out using EQS v.6.4. Reliability was estimated using Cronbach’s alpha, and construct validity was assessed via the Least Squares Robust method. The following goodness-of-fit indices were examined: Satorra-Bentler scaled chi-square (χ²S-B; Satorra & Bentler, 2001); Comparative Fit Index (CFI) and Non-Normed Fit Index (NNFI), with values ≥ 0.90 considered adequate and ≥ 0.95 considered optimal; Root Mean Square Error of Approximation (RMSEA) and Standardized Root Mean Square Residual (SRMR), where values ≤ 0.08 indicate adequate fit and ≤ 0.05 indicate optimal fit (Hu & Bentler, 1999).

Descriptive statistics were then performed using SPSS v.29. Frequencies were calculated for the four identified cybervictimisation trajectories: non-cybervictims, T1-cybervictims, T2-cybervictims, and long-term cybervictims (i.e., those reporting cybervictimisation at both T1 and T2). Classification was based on the cut-off criteria established by the ECIP-Q (Ortega-Ruiz et al., 2016), which defines involvement in cybervictimisation as experiencing any form of cyber-aggression ‘at least once or twice a month’ in the last two months.

To explore the role of peer pressure and anger dysregulation in these trajectories, one-way ANOVAs were performed, followed by Bonferroni-corrected post hoc tests to control for Type I error (see Table 1).

**Table 1:** Means, standard deviations and one-way analyses of variance of anger dysregulation and peer pressure among non-cybervictims, T1-cybervictims, T2-cybervictims and long-term cybervictims (T1T2-cybervictims)

Measure	Non-cybervictims (*n* = 322)	T1 cybervictims (*n* = 22)	T2 cybervictims (*n* = 31)	Long-term cybervictims (*n* = 14)	F(3,389)	η^2^
	*M(SD)*	*M(SD)*	*M(SD)*	*M(SD)*		
T1-ADR	2.41(0.83)	2.93(1.19)	2.54(0.65)	2.93(1.19)	4.11***	0.03
T1-PP	0.65(0.73)	0.91(0.95)	0.89(0.86)	1.12(0.78)	3.01*	0.02
T2-ADR	2.46(0.83)	2.59(0.83)	2.72(0.80)	3.06(0.92)	2.83*	0.02
T2-PP	0.65(0.66)	0.89(0.73)	1.03(0.69)	1.33(0.81)	7.45***	0.05

*Note*. *** *p* < 001; * *p* <.05

Abbreviations: ADR, anger dysregulation; PP, peer pressure

Bivariate correlations were then computed between all study variables, including age. Additionally, independent samples t-tests were run to compare means between boys and girls (see Tables 2 and 3). The gender category “other” (*n* = 1; 0.2%) was excluded from statistical analyses due to insufficient representation.

**Table 2:** Descriptive analyses and correlations for the study variables

	Variable	1	2	3	4	5	6	7
1.	T1-CV	-						
2.	T2-CV	0.39***	-					
3.	T1-ADR	0.23***	0.21**	-				
4.	T2-ADR	0.08	0.22***	0.51***	-			
5.	T1-PP	0.27***	0.23***	0.37***	0.21***	-		
6.	T2-PP	0.23***	0.31***	0.28***	0.37***	0.48***	-	
7.	Age	0.10*	0.04	−0.03	−0.12*	0.09	0.05	-

*Note*. * *p* <.05; ** *p* <.01; *** *p* <.001; Cell entries are zero-order Spearman correlation coefficients. Abbreviations: CV, cybervictimisation; ADR, anger dysregulation; PP, peer pressure

**Table 3:** Descriptive analyses and Student’s t-tests by gender

	M	SD		M	SD	t	*p*	d
1. T1-CV	0.14	0.28	Boys	0.15	0.29	–1.10	0.27	0.11
			Girls	0.12	0.27			
2. T2-CV	0.15	0.27	Boys	0.14	0.24	–1.14	0.26	–0.11
			Girls	0.16	0.29			
3. T1-ADR	2.47	0.83	Boys	2.37	0.80	–2.33	0.02*	–0.24
			Girls	2.58	0.89			
4. T2-ADR	2.52	0.84	Boys	2.45	0.82	–1.57	0.12	–0.16
			Girls	2.59	0.86			
5. T1-PP	0.71	0.76	Boys	0.75	0.77	1.14	0.26	0.12
			Girls	0.66	0.73			
6. T2-PP	0.72	0.69	Boys	0.77	0.66	1.33	0.18	0.13
			Girls	0.67	0.72			

*Note*. * Statistically significant differences *p* <.05

Abbreviations: CV, cybervictimisation; ADR, anger dysregulation; PP, peer pressure

To ensure the adequacy of our sample for the main analyses, we first conducted a power analysis using G*Power. Given that our moderated moderation model involves a three-way interaction (second-order) within a multiple linear regression framework, we followed Cohen’s (1988) guidelines. Parameters were set at a statistical power of 0.95 and a significance level of α = 0.05. With three predictors (one independent variable and two moderators), the analysis indicated that a minimum of 107 participants was required to detect a medium effect size. Our sample exceeded this threshold, and thus was deemed sufficient for the planned analyses.

Finally, a moderated moderation model was tested using PROCESS macro v.4.0 for SPSS (Model 3; Hayes, 2013), employing a longitudinal design (see Fig. 1). The model examined whether peer pressure and anger dysregulation at T2 moderated the longitudinal association between cybervictimisation at T1 (independent variable) and T2 (dependent variable). Age and gender (0 = boys, 1 = girls) were included as covariates (see Fig. 2). Analyses used mean-centred variables, 10,000 bootstrapped samples (95% confidence interval – CI), and HC3 heteroscedasticity-consistent standard errors (Davidson & MacKinnon, 1993). The Johnson-Neyman technique was applied to probe interaction effects, with significance determined at *p* <.05 when the 95% confidence interval excluded zero in the lower or upper limit.

## Results

### Preliminary Analysis

Of the total sample, 5.6% were only T1-cybervictims, 8.0% were only T2-cybervictims, and 3.6% were long-term cybervictims (i.e., T1T2-cybervictims). The analysis of variance showed statistically significant differences between non-cybervictims vs. T1-cybervictims in baseline anger dysregulation (*M* = 2.41 vs. *M* = 2.93; *p*_*bonf*_*=* 0.045), and between non-cybervictims vs. T2-cybervictims (*M* = 0.65 vs. *M* = 1.03; *p*_*bonf*_ = 0.002) and long-term cybervictims (*M* = 0.65 vs. *M* = 1.33; *p*_*bonf*_ = 0.016) in later peer pressure (see Table 1). The effect size was small in all cases, with eta squared > 0.01 and < 0.06 (Cohen, 1988).

### Descriptive Results Overall and by Gender and Age

The descriptive results can be found in Tables 2 and 3. In the overall sample, all variables under study were significantly and positively correlated in each wave, except for cybervictimisation at T1 and anger dysregulation at T2 (*r* =.08, *p* =.120). By gender, the t-test revealed significant differences only in anger dysregulation at T1 (*M*_girls_ = 2.58 vs. *M*_boys_ = 2.37; *p* =.02). In other words, girls scored significantly worse than boys on anger regulation at baseline. By age, the bivariate correlation was only significant for cybervictimisation at T1 (*r* =.13, *p* =.01) and anger dysregulation at T2 (*r* = −.12, *p* =.02). In other words, older adolescents showed significantly higher levels of cybervictimisation at T1, and younger adolescents showed significantly higher levels of anger dysregulation at T2.

### Model Results: Main and Moderating Effects

The model shown in Fig. 2 was statistically significant, *F*(9,378) = 10,44, *p* <.001, *R*^2^ = 0.21. This accounts for 21% of the variance, indicating a medium-sized effect. Neither gender (*β* = 0.05, *p* =.074) nor age (*β* = 0.01, *p* =.625) were found to play a significant role on cybervictimisation at T2.

***Direct Effects***. As shown in Table 4, the ordinary least squares (OLS) regression analyses revealed that cybervictimisation at T1 [*β* = 0.43, *t*(9,378) = 3.49, *p* <.001] and peer pressure [*β* = 0.01, *t*(9,378) = 2.91, *p* <.01] had a significant direct effect on cybervictimisation at T2. In contrast, anger dysregulation [*β* = 0.02, *t*(9,378) = 1.04, *p* =.298] had no significant direct effect on cybervictimisation at T2. In other words, high scores for cybervictimisation at T1 and for peer pressure were strongly associated with cybervictimisation at T2. Only research Hypotheses 1 and 2 were supported (see Fig. 2).

**Table 4:** Direct and interaction effects in the moderate moderation model

Effect	B	SE	95% CI	
			LL	UL
T1-CV	0.43***	0.12	0.19	0.67
PP	0.01**	0.03	0.03	0.17
ADR	0.02	0.02	−0.02	0.05
T1-CV x PP	−0.01	0.05	−0.11	0.10
T1-CV x ADR	0.17*	0.08	0.02	0.33
PP x ADR	−0.07**	0.03	−0.12	−0.02
T1-CV x PP x ADR	−0.22*	0.11	−0.43	−0.01
Gender	0.05	0.03	−0.01	0.10
Age	0.01	0.01	−0.02	0.03

*Note*. Analyses performed using the PROCESS macro for SPSS (Model 3; Hayes, 2013). *p <.05, **p <.01, ***p <.001

Abbreviations: CV, cybervictimisation; PP, peer pressure; ADR, anger dysregulation; CI, confidence interval; LL, lower limit; UL, upper limit

***Moderating Effects***. The next set of research hypotheses addressed the moderating role of peer pressure and anger dysregulation in long-term cyberbullying victimisation, i.e. cybervictimisation at T1 and T2 (H4-H6; see Fig. 2). In the OLS model of these indirect effects, the two-way analyses revealed that peer pressure was not a significant moderator of the association between cybervictimisation at T1 and T2 [*β* = −0.01, *t*(9,378) = − 0.11, *p* =.915; see Table 4]. This result does not support research Hypothesis 4 (H4). However, the two-way analyses revealed a significant positive moderation by anger dysregulation in the association between cybervictimisation at T1 and T2 [*β* = 0.17, *t*(9,378) = 2.20, *p* <.05; see Table 4], thereby confirming research Hypothesis 5 (H5). In other words, high scores for anger dysregulation moderated the association between cybervictimisation at T1 and T2. Moreover, two-way analyses revealed a significant negative moderation by anger dysregulation in the association between peer pressure and cybervictimisation at T2 [*β* = −0.07, *t*(9,378) = − 2.83, *p* <.01; see Table 4], thereby confirming research Hypothesis 6 (H6). In other words, low scores for anger dysregulation moderated the association between peer pressure and cybervictimisation at T2.

**Fig. 3:**
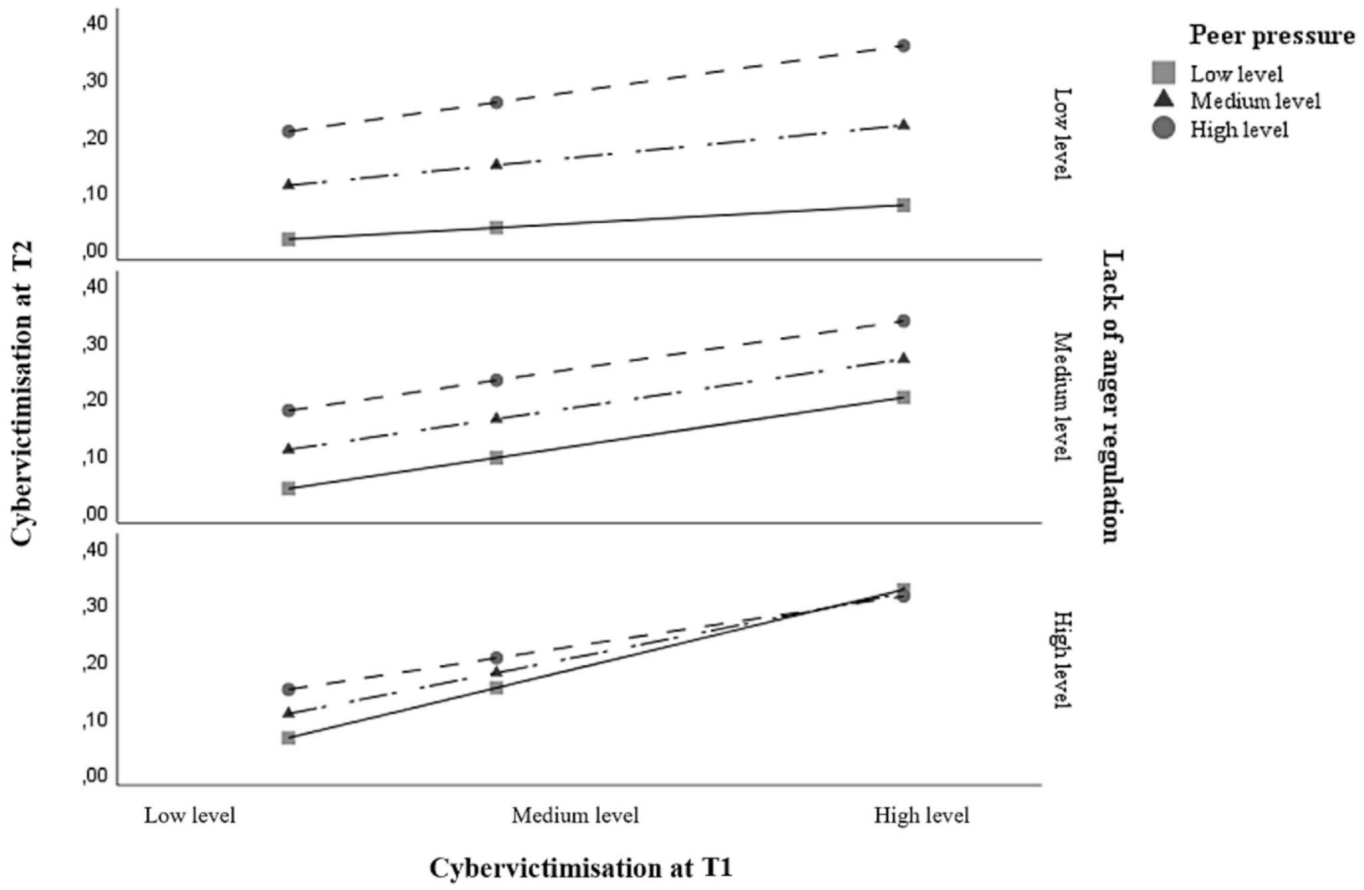
Three-way interaction plot of cybervictimisation at T1, peer pressure, and anger dysregulation on cybervictimisation at T2 *Note*. The moderating effect of anger dysregulation on the conditional influence of peer pressure on the association between cybervictimisation at T1 and T2

By using the overall three-way interaction model, we were able to estimate the moderating effect of anger dysregulation (second moderator) on the conditional influence of peer pressure (first moderator) on the association between cybervictimisation at T1 and T2. In the OLS model of this three-way interaction, the results indicated a negative moderation of peer pressure and anger dysregulation in the association between cybervictimisation at T1 and T2 [*β* = −0.22, *t*(9,378) = − 2.09, *p* <.05; see Table 4]. This supports research Hypothesis 7 (H7; see Fig. 3). Furthermore, the simple slope analysis revealed a significant effect of the cybervictimisation at T1 x peer pressure interaction on cybervictimisation at T2, which was found for high levels of anger dysregulation (*β* = −0.19, *p* <.05; see Fig. 3). In other words, peer pressure acted as a moderator between cybervictimisation at T1 and T2 when anger regulation was not sufficiently developed. As shown in Fig. 3, the lowest level of cybervictimisation at T2 was obtained for the lowest scores of cybervictimisation at T1, peer pressure and anger dysregulation, suggesting that low involvement in previous cybervictimisation and good socioemotional abilities protect against long-term cybervictimisation. In contrast, the highest level of cybervictimisation at T2 was obtained for the highest scores for cybervictimisation at T1, peer pressure and anger dysregulation, suggesting that high involvement in previous cybervictimisation and poor socioemotional abilities increases the risk of long-term cybervictimisation.

## Discussion

Although research on cyberbullying victimisation indicates that most cases tend to resolve over time, a significant proportion persist (González-Cabrera et al., 2023) despite the implementation of effective intervention programmes (Johander et al., 2023), such as ‘ConRed’ (Del Rey et al., 2016) or ‘Cyberprogram 2.0’ (Garaigordobil & Martínez-Valderrey, 2018). Identifying the predictors of long-term cybervictimisation is therefore essential to preventing its most severe and lasting consequences (González-Cabrera et al., 2020; Huang et al., 2022; Tian et al., 2023). This study sought to examine the combined influence of two little-explored socioemotional factors – peer pressure and anger dysregulation – in this context. Gaining insight into their role may help explain why certain cases persist over extended periods (e.g., one year), and could inform the refinement of existing psychoeducational strategies to more effectively support those who are suffering more and for longer.

One of the main contributions of our study is the confirmation that a notable proportion of adolescents experience long-term cybervictimisation – 3.6% of the total sample over a one-year period. This figure represents approximately one-third of those initially identified as cybervictims at T1 (9.2%) and aligns with findings from other Spanish longitudinal studies using similar cut-off points, such as that of Gámez-Guadix and colleagues (2015), with 5.8% of stable cybervictims, or that of González-Cabrera and colleagues (2023), with 6%, of their total samples. This consistency reinforces the idea that cybervictimisation is a dynamic phenomenon, with a significant portion of cybervictims remaining involved over time. Recognising this continuity is crucial: prevalence rates at different time points may reflect the same individuals, not new cases.

In addition, the other key contribution of our study lies in clarifying the potential role of peer pressure and anger dysregulation as predictors of long-term cybervictimisation, both independently and in combination:

In terms of *direct effects*, we confirmed our hypotheses 1 and 2, showing that high levels of prior cybervictimisation and of peer pressure are strong predictors of future cybervictimisation (Camerini et al., 2020; Livazovi’c & Ham, 2019). In this sense, there is sufficient evidence that being a target of cyberbullying at one point in time increases the risk of being targeted again later on (Coelho & Marchante, 2018; González-Cabrera et al., 2023). This may be due, in part, to the fact that the imbalance of power between cybervictims and their cyberbullies and peers becomes increasingly entrenched in social dynamics over time, making it harder to break the cycle of cyberviolence (Chu et al., 2018). Although peer pressure has received less attention in the context of cybervictimisation involvement, our findings highlight its relevance, as Livazovi’c and Ham (2019) or Onditi and colleagues (2024) show cross-sectionally and longitudinally, respectively. Specifically, the increased risk of cybervictimisation among those who feel strongly pressured by their peers may be due to their lower ability to resist peer influence. Individuals who are more impressionable or socially compliant may be perceived as vulnerable by peers, which can lead to social sanctions (Gámez-Guadix et al., 2013), such as a decline in popularity or social preference (Romera et al., 2020). In this respect, susceptibility to peer pressure may act as a marker of social fragility that makes certain adolescents more likely targets. Regarding anger dysregulation (hypothesis 3), our findings did not support its role as a direct predictor of future cybervictimisation. This suggests that emotional reactivity alone may not increase cybervictimisation risk unless it interacts with other factors – such as low social competence or weak peer support networks – that amplify vulnerability.

In terms of *indirect or moderating effects*, our hypotheses 5, 6, and 7 were supported, while, unexpectedly, our hypothesis 4 was not. Specifically, when analysed individually, only anger dysregulation moderated the relationship between prior and future cybervictimisation–indicating that difficulties in managing anger increase the risk of long-term cybervictimisation. The prominent role of emotional factors in long-term cybervictimisation aligns with previous research showing that poor emotional self-control often predicts non-contact victimisation (Pratt et al., 2014). This is the case with cybervictimisation and the use of ineffective coping strategies, which tends to involve adolescents more deeply in the problem (Arató et al., 2022; den Hamer & Konijn, 2016). This may reflect the fact that cyberbullies seek emotional reactions in their cybervictims, provoking them through repeated attacks over time. Our findings thus highlight anger management as a key protective factor – not only to reduce the risk of long-term cybervictimisation but also to prevent maladaptive responses such as rumination (Zsila et al., 2019) or aggression (Camacho et al., 2021).

However, a more nuanced picture of long-term cybervictimisation emerged when examining the interaction between peer pressure and anger dysregulation. In line with our hypothesis 7, the influence of peer pressure appeared to depend on the level of anger dysregulation. Specifically, our three-way interaction analysis revealed that peer pressure alone does not predict long-term cybervictimisation unless considered alongside anger dysregulation levels. For cybervictims with high anger dysregulation, peer pressure did not significantly change their risk of further involvement in the problem – possibly because their emotional vulnerability already places them at high risk. In contrast, for those with moderate or low anger dysregulation (i.e. better emotional self-control), peer pressure did increase the likelihood of long-term cybervictimisation. This finding suggests that even emotionally competent adolescents may remain vulnerable when social influences are strong.

These findings make sense, as we know that anger is an emotion that may lead to greater loneliness (Fang et al., 2020), depression (Huang et al., 2022) and stress (Liu et al., 2020), which in turn heighten the cybervictim’s need to belong (Gao et al., 2021). Cybervictims, who often struggle with social relationships (Romera et al., 2020), may become increasingly afraid of rejection as the cybervictimisation persists (van den Eijnden et al., 2014). In an attempt to regain acceptance, they may give in to peer pressure – adopting certain behaviours or complying with peer norms to fit in (Gao et al., 2021). However, very susceptibility can make them less appealing to their peers, as being overly impressionable is often perceived negatively and associated with lower social status or popularity (Gámez-Guadix et al., 2013; Romera et al., 2020; van den Eijnden et al., 2014). This cycle ultimately isolates cybervictims further, depriving them of peer support that could help protect them from ongoing cybervictimisation (Navarro et al., 2015).

Hence, our study underscores two key socioemotional conditions – peer pressure and anger dysregulation – that may allow cyberbullies to prolong their attacks over time. These findings have direct implications for optimising generic prevention and intervention measures aimed at combating cyberbullying. In particular, it seems crucial to incorporate: (i) emotional regulation (e.g., managing impulsive reactions to online provocation), which may be especially effective for adolescents with high anger dysregulation (den Hamer & Konijn, 2016); and (ii) training in peer resistance skills, assertiveness, and critical thinking about peer norms and behaviors, which may be especially beneficial for those with lower emotional vulnerability (Onditi et al., 2024). Indeed, this is particularly important, as previous research suggests that enhancing adolescents’ emotional regulation strategies can lead to improved social competence, and conversely, stronger social competence can further enhance emotional regulation (Arató et al., 2022). Therefore, based on our findings, we argue that cyberbullying interventions should be integrative, addressing both internal (emotional regulation) and external (peer pressure) risk factors simultaneously, as some adolescents remain at risk of long-term cybervictimisation.

Finally, while our study primarily aimed to provide a general overview of cybervictimisation over time, we included gender and age solely as covariates in our theoretical model. Although previous research has indicated a higher prevalence of cybervictimisation among girls (Festl et al., 2017; Smith et al., 2019) and older adolescents (WHO, 2020), neither gender nor age emerged as significant variables in our analyses. This suggests that, while these factors may play a role in cybervictimisation, they were not central to the scope of our current study. However, further research is needed to examine the moderating role of gender and age in greater depth, as their influence on long-term cybervictimisation may vary across different populations.

### Limitations and Future Research

This study has several limitations. First, it relied on a convenience sample from a specific region of Spain, which restricted comparisons to only boys and girls due to the relatively small sample size. To enhance the generalisability of the findings, it is crucial to expand the sample both nationally and internationally, ensuring greater diversity, especially in terms of gender and other minority groups, who are at increased risk of general or stigma-based cyberbullying (Llorent et al., 2016; Ojeda et al., 2023). Second, our study used exclusively self-report measures, which are susceptible to biases such as social desirability and recall bias. To address this, it would be valuable to incorporate additional methods, such as reports from peers or teachers, as well as observational data, to triangulate the findings. Other qualitative methods, such as focus groups, could also provide richer insights into participants’ experiences and perceptions of difficulties and strategies in dealing with peer pressure or anger dysregulation, which may not have been covered by the items in the measures used. Third, while the one-year longitudinal design offers useful insights, extending the study to include more time points would provide a more comprehensive understanding of response trends and the evolving dynamics of the variables over time. Finally, the study did not assess whether long-term cybervictims were already cybervictimised prior to T1, leaving uncertainty about whether their cybervictimisation was a pre-existing condition or developed during the study period.

Our study also offers several directions for future longitudinal research. First, examining the interactions between socioemotional and contextual variables is crucial for gaining a deeper understanding of cybervictimisation dynamics over time. For instance, while socio-familial support has been shown to help victims cope with cyberbullying (Espino et al., 2023), its potential role in preventing long-term cybervictimisation, similar to its effect on bullying victimisation (Healy et al., 2024), remains underexplored. Investigating how different types of support – e.g. emotional or instrumental – affect resilience to ongoing victimisation could help refine prevention and intervention strategies. In addition, future research should consider the factors that drive the persistence of cyberbullies, such as moral disengagement or belonging needs (Piccoli et al., 2020). Such insights would contribute to a more nuanced understanding of the long-term dynamics of cyberbullying and inform the design of prevention and intervention strategies that go beyond supporting victims to also address the underlying socioemotional mechanisms that perpetuate aggressive behaviour.

### Prevention Implications and Conclusions

Our findings provide clear practical implications. Notably, one in three adolescents who reported being cybervictims at the beginning of the study remained cybervictimised a year later. This continuity highlights the urgent need to better understand and address the mechanisms behind long-term cybervictimisation. In this respect, our study points to peer pressure and anger dysregulation as key socioemotional factors that contribute to the risk of continued cyberbullying over time – both of which are modifiable and can therefore be targeted in prevention and intervention programmes. Hence, our findings offer guidance on which components should be added or reinforced in current and future cyberbullying interventions to enhance their effectiveness, particularly in addressing more complex cases such as long-term cybervictims (Johander et al., 2023). In other words, efforts to raise awareness about cyberbullying and initiatives aimed at enhancing socio-emotional skills should be integrated into a cohesive and comprehensive approach in order to effectively prevent and reduce cyberbullying. This would allow a single programme to address the influence of peer pressure and anger dysregulation in the normalisation of cyberbullying – for instance, through role-playing activities where students practise resisting peer pressure or managing emotional reactions in online peer conflicts.

In conclusion, our findings are useful for educators, school counsellors, families, mental health professionals, and policymakers on a number of critical issues: (i) a significant proportion of cybervictims continue to suffer over time and are at risk of more severe psychological consequences; (ii) there is an urgent need to integrate socioemotional education into cyberbullying prevention and intervention strategies to enhance potential cybervictims’ ability to resist peer pressure and manage anger; and (iii) addressing the role of cyberbullies and cyberbystanders is equally crucial to reduce the social acceptance of violence. These implications call for a more comprehensive, context-sensitive approach to cyberbullying that combines individual support with school-wide and community-level strategies to foster safer and more inclusive peer relationships in physical and virtual contexts.

## Data Availability

The data that support the findings of this study are available from the corresponding author upon reasonable request.
